# Trends in Use of Antiseizure Medicines in Women With Epilepsy in Pregnancy During 2018–2023 in an Irish Maternity Hospital: A Cohort Study

**DOI:** 10.1155/ogi/9975717

**Published:** 2026-09-15

**Authors:** Joan E. Devin, Fergal O’Shaughnessy, Brian J. Cleary, Jennifer C. Donnelly, Nicola Maher

**Affiliations:** ^1^ Irish Medicines in Pregnancy Service, Rotunda Hospital, Parnell Square, Dublin 1, Ireland, rotunda.ie; ^2^ School of Pharmacy and Biomolecular Sciences, RCSI University of Medicine and Health Sciences, Dublin 2, Ireland; ^3^ School of Medicine, RCSI University of Medicine and Health Sciences, Dublin 2, Ireland; ^4^ Department of Obstetrics and Gynaecology, Rotunda Hospital, Parnell Square, Dublin 1, Ireland, rotunda.ie; ^5^ Epilepsy Service, Rotunda Hospital, Parnell Square, Dublin 1, Ireland, rotunda.ie

**Keywords:** antiseizure medicines, epilepsy, pregnancy, prescribing trends, seizure disorders

## Abstract

**Objective:**

This study aimed to describe and explore the trends of antiseizure medicine (ASM) use in women with epilepsy (WWE) in pregnancy over a 6‐year period.

**Methods:**

This observational cohort study used routinely collected electronic health record (EHR) data from a large maternity hospital in Dublin, Ireland, with > 9000 births annually. WWE attending during pregnancy between 2018 and 2023 were included. Descriptive statistics and linear and negative binomial regression models were used to assess temporal trends. Outcomes included individual ASM exposure, ASM safety category (‘Known safest’, ‘Uncertain risk’ and ‘Higher risk’) and temporal trends in ASM use.

**Results:**

There were 286 ASM‐exposed pregnancies in WWE. The overall proportion of ASM‐exposed pregnancies in WWE ranged between 87.7% and 95.7% per year. Trends for most ASMs and overall safety categories did not change significantly or had insufficient data to assess trends over time. Levetiracetam (60.1%) and lamotrigine (31.5%) were most frequently used overall. Zonisamide use decreased significantly (−0.395, *p* = 0.029), although this finding should be interpreted cautiously given the small number of exposures (*n* = 13). ASM polytherapy occurred in 24.8% of pregnancies. Some newer ASMs were introduced during the study, and a decline in use of some higher‐risk ASMs was observed, although findings were not significant.

**Conclusions:**

In this single‐centre EHR cohort, trends in use of ASMs in WWE appeared relatively stable, although analyses of less commonly used ASMs were limited by very small numbers. ASMs with safer pregnancy profile were most frequently used in WWE in pregnancy. Additional data are needed to further assess trends and outcomes and to determine the place of newer generation ASMs in WWE in pregnancy.

## 1. Introduction

Epilepsy is the most common serious neurological disorder seen in pregnant women [1, 2], affecting an estimated 0.3%–1.0% of pregnancies globally each year [3–5]. Epilepsy in pregnancy can lead to substantial maternal and neonatal morbidity and mortality and is one of the most frequent causes of indirect maternal death [1, 5, 6].

Untreated or poorly controlled epilepsy in pregnancy may lead to seizure activity and adverse maternal and neonatal outcomes; therefore, effective seizure control, where necessary, using antiseizure medicines (ASMs) is key [1]. It is now established that ASMs differ in their safety profile for use in pregnancy, and it is advised that women with epilepsy (WWE) switch to an ASM with a safer risk profile which maintains seizure control preconception, unless other treatments are ineffective or not tolerated [7–9]. Levetiracetam and lamotrigine are considered the safest choice for use in pregnancy [8–10], while the risk of other ASMs, for which there are fewer data, is uncertain [9, 10]. The use of sodium valproate, topiramate and phenobarbital in pregnancy is associated with increased risk of congenital malformations, growth restriction or adverse neurodevelopmental outcomes [6, 7, 9, 10]. Newer ASMs are increasingly prescribed for WWE where seizure control is not achieved with existing therapies or who cannot tolerate other ASMs due to adverse effects, but published data on their use are limited [10–12].

Limited information is available on the prevalence and patterns of ASM use in pregnancy in different jurisdictions. Previous Irish research identified that while valproate prescribing decreased in WWE between 2008 and 2013, prescribing for other indications appeared to increase [13]. Kinney et al. [14] similarly found that use of valproate and carbamazepine decreased between 1996 and 2016 in the United Kingdom and Ireland. Other studies have focused on safety and outcomes related to particular ASMs, such as zonisamide [15].

Most data on ASM use in WWE in Ireland are derived from the UK and Irish Epilepsy and Pregnancy Register, a prospective, observational, registration and follow‐up study that was set up in 1996 to determine the relative safety of all ASMs used in pregnancy [7, 16]. As registries rely on voluntary enrolment, they may not capture all ASM exposures in pregnancy. The use of electronic health records (EHRs) for research purposes may provide additional insight into trends in prescribing on a larger scale, using routinely collected healthcare data.

This study aimed to describe and explore the prevalence and trends of ASM use in WWE in pregnancy in an Irish maternity hospital between 2018 and 2023.

## 2. Methods

This was an observational cohort study that examined trends in use of ASM in WWE in pregnancy using routinely collected data from the Maternal and Newborn Management System (MN‐CMS) EHR. The Strengthening the Reporting of Observational Studies in Epidemiology (STROBE) guidelines were used for reporting (see Supporting File 1) [17]. There was no patient or public involvement in this study.

### 2.1. Study Setting and Population

The Rotunda Hospital is a large maternity hospital in Dublin, Ireland, with over 9000 births annually. WWE attend the obstetric‐led multidisciplinary epilepsy clinic, which maintains close links with neurology services.

Women with a diagnosis of active epilepsy and who attended the Rotunda Hospital in pregnancy 2018–2023 were included in this study. All eligible pregnancies in WWE during the study period were included, and no formal power calculation was performed as the analysis was based on the complete available cohort. A woman could contribute > 1 pregnancy. Only women with completed pregnancies up to 2023 were included; those with ongoing pregnancies were excluded. Women with a history of epilepsy but who did not meet the criteria for an active epilepsy diagnosis (e.g., childhood epilepsy or seizure‐free > 10 years while medication‐free) were also excluded from the study.

### 2.2. ASM Exposure

Epilepsy diagnoses in the EHR are captured through data recorded by midwives at the antenatal booking interview and recorded as structured diagnoses. ASM orders for the study period were retrieved from the EHR using in‐built reporting functions and cross‐referenced by the antenatal booking diagnosis to identify WWE with ASM‐exposed pregnancies. A diagnosis of active epilepsy, as per ILAE classification [18], was confirmed through chart review. Prescriptions, inpatient medication orders and medication history documentation were used to ascertain exposure to individual ASMs in pregnancy. ASM exposure was defined as at least one order for an ASM at any stage during pregnancy. Polytherapy was defined as a record of ≥ 2 unique ASMs in pregnancy (see S1, Supporting File 2, for full ASM search list). Due to the nature of the available EHR data, it was not possible to distinguish concurrent use from sequential monotherapy (e.g., switching between ASMs during pregnancy), nor to determine overlap in treatment duration. Timing of exposure by trimester was not consistently available for all prescriptions; therefore, analyses reflect ASM exposure at any time during pregnancy rather than trimester‐specific exposure.

ASM prescriptions were further classified by safety profile in pregnancy into three categories, using criteria previously described by Houben et al. [11]. The ‘Known safest’ category included lamotrigine and levetiracetam. The ‘Uncertain risk’ category included brivaracetam, clobazam, clonazepam, eslicarbazepine, ethosuximide, gabapentin, lacosamide, oxcarbazepine, perampanel, pregabalin and zonisamide. The ‘Higher risk’ category included carbamazepine, phenobarbital, sodium valproate and topiramate.

### 2.3. Maternal and Obstetric Characteristics

Maternal and obstetric characteristics of interest were retrieved from the EHR using in‐built reporting functions and included age at delivery, year of delivery, parity, ethnicity, body mass index (BMI), birth outcome, gestational age at delivery and delivery type. Type of care (e.g., public, private and semiprivate) was used as a proxy for socioeconomic status. Missing variables, if available, were retrieved by J.E.D. using retrospective chart review.

### 2.4. Statistical Analysis

Descriptive statistics were used to summarise demographic and clinical characteristics for WWE with ASM‐exposed and non–ASM‐exposed pregnancies. Trends in ASM use over time were analysed for each individual ASM and by overall safety categories. Temporal trends were analysed using linear regression for continuous data and negative binomial regression for count data, with year of delivery modelled as a continuous predictor. Negative binomial regression was used to account for overdispersion in count data. Models were unadjusted for confounders, as the intention was to describe temporal trends and not causal relationships. An exploratory analysis of interaction terms between safety profile and year was conducted to assess whether trends differed between safety profile groups. Regression coefficients are presented with 95% confidence intervals (CIs). Statistical inference was based on Wald tests. All tests were two‐sided, and a *p* value of < 0.05 was considered statistically significant. Analyses were conducted using *R* Version 4.3.0 [19].

## 3. Results

There were 311 pregnancies identified in WWE out of a total 60,312 pregnancies in the Rotunda Hospital during 2018–2023, with 40 women contributing > 1 pregnancy. Of these 311 pregnancies, 286 (92.0%) were exposed to ASMs, with just 25 (8.0%) unexposed to ASMs.

A further 111 women disclosed a history of epilepsy at their booking appointment and received a once‐off consultation with the Epilepsy Clinic, but they did not meet the criteria for an active epilepsy diagnosis (e.g., childhood epilepsy or seizure‐free > 10 years while medication‐free) and so were excluded from this study.

Demographic characteristics and descriptive statistics for both ASM‐exposed and non–ASM‐exposed pregnancies in WWE are summarised in Table 1. Mean age was slightly higher in the ASM‐exposed group (32 years, SD 6) than in the non–ASM‐exposed group (29 years, SD 7), with most WWE in both groups aged between 31 and 40 years during pregnancy. A higher proportion of the non–ASM‐exposed group than the ASM‐exposed group was nulliparous at booking (60.0% vs. 45.1%). Similar proportions of women in both groups received private or semiprivate care.

**TABLE 1:** Maternal and obstetric characteristics of pregnancies in WWE, 2018–2023.

Characteristic	ASM‐exposed pregnancies *N* = 286, *n* (%)	Non‐ASM‐exposed pregnancies *N* = 25, *n* (%)
Age at delivery (years)		
≤ 20	9 (3.1)	3 (12.0)
21–30	100 (35.0)	9 (36.0)
31–40	165 (57.7)	12 (48.0)
41–49	12 (4.2)	1 (4.0)
Mean ± SD	32 ± 6	29 ± 7
Care pathway		
Public	222 (79.6)	20 (80.0)
Private/SPC	64 (22.4)	5 (20.0)
Ethnicity		
White Irish	217 (75.9)	20 (80.0)
Irish Traveller	5 (1.7)	2 (8.0)
Other White background	31 (10.8)	1 (4.0)
Black/Black Irish	3 (1.0)	0 (0)
Asian/Asian Irish	6 (2.1)	1 (4.0)
Other/Mixed background	14 (4.9)	1 (4.0)
Unknown	10 (3.5)	0 (0)
BMI		
Mean ± SD	26 ± 6	28 ± 7
Parity (before delivery)		
0	129 (45.1)	15 (60.0)
1	90 (31.5)	6 (24.0)
2	44 (15.4)	3 (12.0)
≥ 3	23 (8.1)	1 (4.0)
Pregnancy outcome^a^		
Live birth	272 (94.8)	24 (92.3)
Miscarriage	8 (2.8)	0 (0)
Stillbirth	2 (0.7)	0 (0)
Neonatal death	1 (0.3)	2 (7.7)
Unknown	4 (1.4)	0 (0)
GA at birth (weeks)^a^, ^b^		
< 28	1 (0.4)	2 (7.7)
28–< 32	5 (1.9)	1 (3.8)
32–< 37	27 (9.7)	0 (0)
37– ≤ 40	231 (84.0)	18 (69.2)
> 40	11 (4.0)	5 (19.2)
Median [IQR]	39 [2]	39 [2]
Birth weight (g)^a^, ^b^		
< 1000–1499	4 (1.4)	2 (7.7)
1500–2499	22 (8.0)	1 (3.8)
2500–4499	247 (89.8)	22 (84.6)
≥ 4500	1 (0.4)	0 (0)
Unknown	1 (0.4)	1 (3.8)
Median [IQR]	3300 [738]	3480 [790]
Delivery type		
SVD	116 (40.6)	10 (40.0)
OVD	39 (13.6)	7 (28.0)
C‐section	122 (42.7)	7 (28.0)
Unknown	9 (3.1)	1 (4.0)

Abbreviations: ASM, antiseizure medicines; BMI, body mass index; C‐section, caesarean section birth; g, grammes; GA, gestational age; IQR, interquartile range; OVD, operative vaginal delivery/birth; SD, standard deviation; SVD, spontaneous vaginal delivery/birth; WWE, women with epilepsy.

^a^Both ASM‐exposed and non–ASM‐exposed births included 1 multiple birth.

^b^Excludes miscarriages and unknown outcomes.

The rate of live birth in the ASM‐exposed group was 94.8%, while in the non–ASM‐exposed group it was 92.3%. The rate of miscarriage was 2.8% in the ASM‐exposed group, while there were none in the non–ASM‐exposed group. The majority of babies in both groups were born at term and were within the normal birth weight range (2500–4499 g).

### 3.1. Trends in ASM Use

#### 3.1.1. Trends in Use of Individual ASMs

**FIGURE 1:**
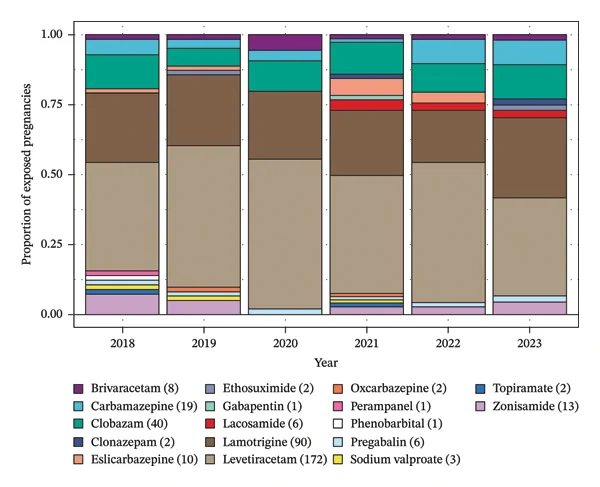
Bar chart of ASM use in WWE in pregnancy, 2018–2023.

During the study period, overall ASM prescribing among WWE ranged between 87.7% and 95.7%, with a total of 17 different ASMs identified (Figure 1). There were a low number of exposures to ASMs that are also used for indications other than epilepsy, such as pregabalin and gabapentin. The proportion of WWE using ASMs did not change significantly over time.

Levetiracetam and lamotrigine were the most frequently prescribed ASMs between 2018 and 2023, used in 60.1% and 31.5% of ASM‐exposed pregnancies, respectively. Clobazam was used in 14.0% of pregnancies. Other ASMs used less frequently were carbamazepine (6.6%), zonisamide (4.5%), eslicarbazepine (3.5%), brivaracetam (2.8%), lacosamide (2.1%), pregabalin (2.1%) and sodium valproate (1.0%). Eleven pregnancies (3.8%) were exposed to clonazepam, ethosuximide, oxcarbazepine, topiramate, gabapentin, perampanel or phenobarbital. Exposures from women who contributed > 1 pregnancy did not disproportionately affect the rates of less common ASM exposures in the dataset, with the majority of these women exposed to levetiracetam and lamotrigine. See Figure S1, Supporting File 2, for linear trends over time for the eight of the most prevalent ASMs used in WWE in pregnancy.

A linear regression model was used to analyse individual ASMs with use in ≥ 2 years. Based on 13 total exposures, zonisamide was observed to have a significant negative trend over time (−0.395, *p* = 0.029), with a good model fit (*R* ^2^ = 0.840), indicating that its use in WWE in pregnancy decreased significantly between 2018 and 2023. However, given the small number of exposures, findings are limited and should be interpreted with caution. The models for all other ASMs showed no significant trends over time or had insufficient variation in the data to produce a meaningful model. Univariate linear regression analyses are provided in Table S2, Supporting File 2.

#### 3.1.2. Trends in Use by ASM Safety Profile

**FIGURE 2:**
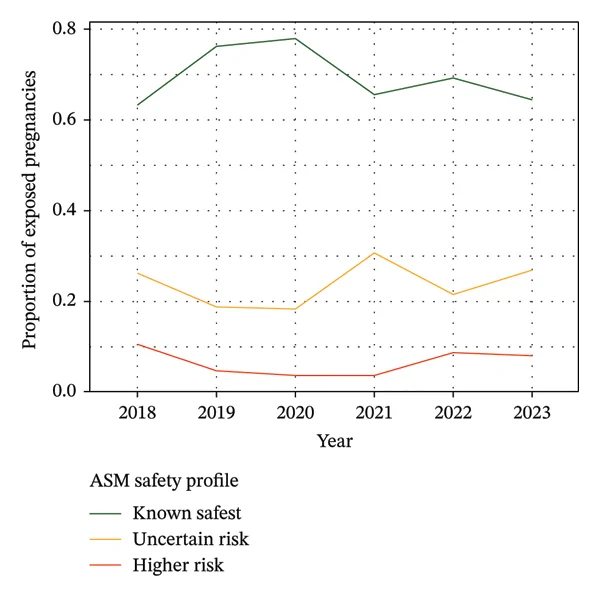
Trends of ASM use in WWE in pregnancy by safety risk category, 2018–2023.

Figure 2 displays trends over time of ASM use in WWE in pregnancy by safety risk category (‘Known safest’, ‘Uncertain risk’ and ‘Higher risk’) during the study period. Negative binomial regression, suitable for overdispersed count data, found no significant association between ASM safety risk category and year of delivery, indicating that prescribing patterns remained stable across safety profiles (Table 2). Using the ‘Higher risk’ category as reference, the coefficients were 0.071 (standard error [SE] 0.209, 95% CI −0.318 to 0.506, *p* = 0.734) for ‘Known safest’ and 0.234 (SE 0.226, 95% CI –0.192 to 0.697, *p* = 0.300) for ‘Uncertain risk.’ The intercept estimate had a large SE, reflecting considerable uncertainty, which was likely due to sparse data in some categories. Coefficients represent the change in the log‐expected yearly count of deliveries relative to the ‘Higher risk’ group.

**TABLE 2:** Negative binomial regression analysis of ASM safety risk category by year of delivery.

	Estimate (95% CI)	Std. Error	Z‐value	*p* value
Higher risk (intercept)	0.052 (−125.297, 124.829)	63.78	0.001	0.999
Known safest	0.071 (−0.318, 0.506)	0.209	0.340	0.734
Uncertain risk	0.234 (−0.192, 0.697)	0.226	1.037	0.300

Abbreviations: ASM, antiseizure medicines; CI, confidence interval; std. error, standard error.

While the model demonstrated no significant change in the use of ASMs by overall safety profile, there was some variation in the ASMs within the ‘Uncertain risk’ category and ‘Higher risk’ category between 2018 and 2023.

The first exposure to lacosamide occurred in 2021, as use of other ASMs in the ‘Uncertain risk’ category decreased, including perampanel, gabapentin, ethosuximide and oxcarbazepine (Figure 1). Use of clobazam and brivaracetam appeared consistent, as there were pregnancies exposed to both of these ASMs each year during the study period. Zonisamide‐exposed pregnancies were identified in all years except 2020, despite the decreasing trend in its use.

In 2018, there were pregnancies exposed to all four ASMs in the ‘Higher risk’ category (carbamazepine, phenobarbital, sodium valproate and topiramate). By 2023, only carbamazepine‐exposed pregnancies were identified, as use of the other ASMs in this category decreased. The most recent exposure to sodium valproate or topiramate in pregnancy in this cohort of WWE occurred in 2021 (Figure 1).

### 3.2. ASM Polytherapy

**FIGURE 3:**
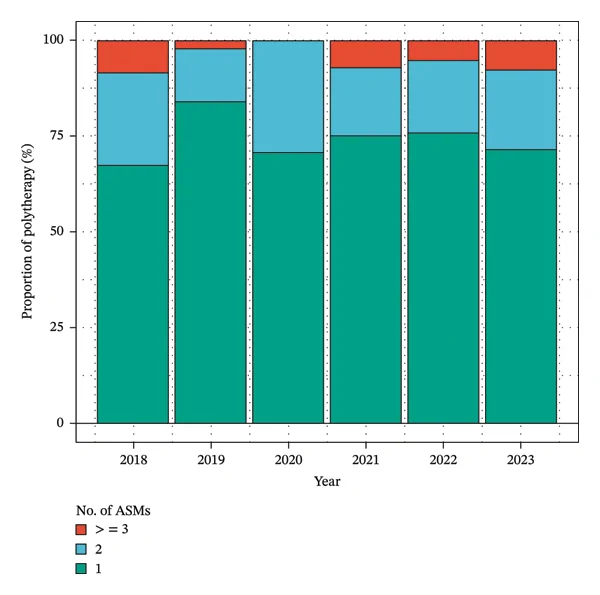
Proportion of polytherapy in ASM‐exposed pregnancies.

ASM monotherapy was more common than polytherapy regimens across all years of the study, with 75.2% of WWE who were exposed to ASMs in pregnancy taking one ASM. The overall proportion of WWE taking two ASMs and three or more ASMs was 19.9% and 4.9%, respectively (see Table S1, Supporting File 2), although it was not possible to distinguish concurrent use from sequential monotherapy, nor to determine overlap in treatment duration. The overall proportion of polytherapy in ASM‐exposed pregnancies remained relatively stable during the study period, with no significant changes observed (Figure 3). Levetiracetam was used in 70.4% of polytherapy regimens. Clobazam was used in 52.1% polytherapy regimens, potentially reflecting its use as adjunct therapy when undertaking adjustments and titrations of other ASMs. Other ASMs used in polytherapy included lamotrigine (45.0%), zonisamide (12.7%) and carbamazepine (7.9%) (Table 3). There was monotherapy only in the two pregnancies exposed to perampanel or phenobarbital.

**TABLE 3:** Proportion of ASM used in polytherapy regimens.

	Pregnancies with ASM polytherapy *N* = 71 *n* (%)
*ASM*	
Levetiracetam	50 (70.4)
Clobazam	37 (52.1)
Lamotrigine	32 (45.0)
Zonisamide	9 (12.7)
Carbamazepine	6 (7.9)
Eslicarbazepine	4 (5.6)
Brivaracetam	4 (5.6)
Lacosamide	4 (5.6)
Pregabalin	4 (5.6)
Sodium valproate	2 (2.8)
Clonazepam	2 (2.8)
Ethosuximide	1 (1.4)
Oxcarbazepine	1 (1.4)
Topiramate	1 (1.4)
Gabapentin	1 (1.4)
*No. of concurrent ASMs*	
2	57 (19.9)
≥ 3	14 (4.9)

Abbreviation: ASM, antiseizure medicines.

## 4. Discussion

### 4.1. Main Findings

There were 311 pregnancies in WWE during 2018–2023, with 286 (92.0%) exposed to ASMs. Levetiracetam and lamotrigine were the most frequently used ASMs. Other ASMs used less frequently included clobazam, carbamazepine, zonisamide, eslicarbazepine, brivaracetam, lacosamide, pregabalin, clonazepam, ethosuximide, oxcarbazepine, topiramate, gabapentin, perampanel and phenobarbital. There were three exposures to sodium valproate.

Findings suggest that use of most ASMs remained relatively stable or decreased during the study period. Due to small numbers of individual ASMs or insufficient variation in the data, most ASMs did not demonstrate significant changes in use, except for zonisamide, which demonstrated a negative trend in use between 2018 and 2023. A negative binomial regression model examining change of use in ASM by risk category (‘Known safest’, ‘Uncertain risk’ and ‘Higher risk’) did not identify significant changes in use by year. However, there was some variation in the distribution of ASMs within the safety categories during the study period, with no use of phenobarbital observed after 2018, and no use of sodium valproate or topiramate observed after 2021 in the ‘Higher risk’ category.

Twenty‐five WWE (8.0%) did not take ASMs in pregnancy in this study. Although further examination of this cohort and the individual reasons for lack of ASM exposure was outside the scope of this study, this small number is likely representative of the low numbers of WWE who choose not to take ASMs in pregnancy.

### 4.2. Interpretation

#### 4.2.1. Trends in Use of ASM

Relatively stable trends for the individual ASMs in this study suggest that ASMs with safer pregnancy profiles are well established in clinical practice in the Rotunda Hospital and affiliated neurology services. Levetiracetam and lamotrigine were the two most frequently used ASMs in this study across all years between 2018 and 2023, exceeding the use of other ASMs by a large margin. This is in line with Kinney et al. [14], who previously found statistically significant increases in lamotrigine and levetiracetam prescribing in WWE in the UK and Ireland Epilepsy and Pregnancy Register between 1996 and 2016. The overall rate of monotherapy in our study (75.2%) was also similar to the overall rate identified in the register (73.4%) [14].

It is known that the type of ASM included in polytherapy is at least as important as the number of ASMs when considering risks to the fetus [2]. Levetiracetam was the most common ASM in polytherapy‐exposed pregnancies. The benzodiazepines clobazam and clonazepam were used almost exclusively in polytherapy regimens, potentially reflecting their intermittent use as adjunct therapy when undertaking adjustments and titrations. While a small proportion of pregnancies (4.9%) were exposed to three or more ASMs, exposures may not have been concurrent.

In September 2023, new pregnancy prevention programme (PPP) recommendations were issued in relation to topiramate for epilepsy (but not migraine prophylaxis) to reduce the risks of congenital malformations, small for gestational age (SGA) and neurodevelopmental delay now shown to be associated with maternal topiramate use in pregnancy. Restricted use of sodium valproate in girls and women of childbearing age was recommended in 2014, and a PPP was subsequently introduced in 2018. The last documented exposed to topiramate in WWE occurred before the introduction of the PPP; however, use for migraine prophylaxis was not considered in this study.

There were three exposures to sodium valproate during the study period. There are exceptional circumstances in which these agents may be necessary in pregnancy, as in some WWE other ASM may be ineffective or not tolerated [9]. Stopping therapy in some cases may confer considerable risk to both the mother and the fetus, including increased seizures, status epilepticus and sudden unexpected death in epilepsy (SUDEP) [20, 21], and a woman who is fully informed about the risks of sodium valproate in pregnancy may still choose to remain on treatment [20]. Hughes et al. [22] identified a significant decline in the prevalence ratio of sodium valproate use in Ireland after 2018, and low overall numbers indicate minimal use of sodium valproate in our cohort, with exceptional circumstances or unintended pregnancy being potential reasons for its use.

The use of newer‐generation ASMs in pregnancy is thought to be increasing as use of higher‐risk medications declines, particularly in women who require polytherapy for seizure control or who have a poor response to known safe medications [11, 12]. There was no significant increase in the use of newer generation ASMs in our study due to overall small numbers, although some changes were observed. The first exposure to lacosamide occurred in 2021, while the proportion of eslicarbazepine‐exposed pregnancies was higher in 2021 and 2022 than in previous years. Brivaracetam use appeared consistent, which may be reflective of it primarily being used in WWE who have experienced a low mood with levetiracetam.

#### 4.2.2. Zonisamide

There were 13 pregnancies exposed to zonisamide in the study, with a decline in use observed between 2018 and 2023. This may be due to prescriber or maternal preference for ASMs with more information in pregnancy or increasing concerns around potential teratogenicity and growth restriction arising from exposure in pregnancy [15, 23]. Although data on zonisamide use during pregnancy are limited, studies from the North American, the United Kingdom and Ireland registers have reported SGA rates ranging between 15% and 21% [15, 23]. Exposure data from the United Kingdom, Ireland and Australian registers have also raised concerns about the potential teratogenicity of zonisamide, particularly in higher doses, but further studies are needed to evaluate risks [15, 24]. Given the history of ASMs in pregnancy, prescribers may be more responsive to potential signals and change their prescribing habits than for medicines for other conditions. Although we did not focus on outcomes in this study, just one (7.7%) of the 13 neonates exposed to zonisamide in pregnancy had a low birth weight (< 2500 g) at term.

### 4.3. Strengths and Limitations

To our knowledge, this is the first study that looks at trends in use of ASMs in pregnancy in WWE in Ireland using EHR data. Strengths of this study include the use of EHR data from a maternity hospital that serves a large proportion of WWE in Ireland. We reported demographics and outcomes for ASM‐exposed and non–ASM‐exposed WWE who attended the hospital during the study period. ASMs were categorised using established safety profiles. All women included in this study had a known diagnosis of active epilepsy, and there were a low number of exposures to ASM that are also used for indications other than epilepsy, such as pregabalin and gabapentin.

Although we defined exposure as the presence of any ASM order in pregnancy, it was not possible to determine adherence in this cohort. Reports from an EHR used for maternity care may not capture primary care or neurology prescriptions and cannot determine timing of ASM initiation or discontinuation. It was not possible to capture ASM dose changes or duration of treatment, as this information was frequently documented in unstructured data. However, as medication exposure is documented in structured form at various time points in pregnancy in the EHR, ascertainment of ASM exposure itself is likely conclusive. The definition of polytherapy assumes that multiple ASMs recorded throughout the pregnancy were used concurrently, which may not be the case. As such, this definition likely overestimates rates of polytherapy and also potentially obscures critical safety differences between first‐trimester ASM exposure and later exposure. Many individual ASMs had low exposures overall, making it difficult to adequately assess trends for these drugs without more data.

Exposed pregnancies in this study were limited to those where hospital care was provided, meaning that spontaneous miscarriages which occurred prior to the booking visit (approximately 12 weeks’ gestation) and early terminations were not routinely captured, introducing the potential for selection bias. Pregnancies exposed to ASMs associated with an increased risk of pregnancy loss or severe fetal anomalies may be incompletely captured and therefore be underrepresented, potentially leading to underestimation of adverse pregnancy outcomes for some ASMs. Similar limitations have been previously described [25]. However, given the retrospective observational study design, trends in ASM use in this cohort cannot be inferred from these data.

## 5. Conclusions

The proportion of ASM‐exposed pregnancies in WWE attending the Rotunda Hospital between 2018 and 2023 was high, at 92.0% overall. Levetiracetam and lamotrigine, ASMs with a safer‐use profile, were most frequently used. Approximately 25% of pregnancies involved ASM polytherapy. Trends in use of individual ASMs appeared relatively stable or declined. Zonisamide use decreased significantly, although this finding should be interpreted cautiously given the very small number of exposures. As small numbers limited analysis, further data are needed to assess trends and outcomes and to determine the place of newer generation ASMs in WWE in pregnancy.

## Author Contributions

Joan E. Devin: conceptualisation, methodology, formal analysis, investigation, project administration and writing–original draft. Fergal O’Shaughnessy: conceptualisation, methodology and writing–review and editing. Brian J. Cleary, Jennifer C. Donnelly and Nicola Maher: conceptualisation and writing–review and editing.

## Funding

No funding was provided for this project.

## Disclosure

Joan E. Devin affirms that this manuscript is an honest, accurate, and transparent account of the study being reported; that no important aspects of the study have been omitted; and that any discrepancies from the study as planned (and, if relevant, registered) have been explained.

All authors have read and approved the final version of the manuscript. Joan E. Devin had full access to all of the data in this study and takes complete responsibility for the integrity of the data and the accuracy of the data analysis.

## Ethics Statement

This study was approved by the Rotunda Hospital Research Advisory Group on 25 March 2024 (RAG‐2024‐007). As a low‐risk, retrospective chart review using deidentified data, the approving REC determined that individual participant consent was not required. All data were handled in accordance with relevant data protection guidelines.

## Conflicts of Interest

The authors declare no conflicts of interest.

## Supporting Information

Additional supporting information can be found online in the Supporting Information section.

## Supporting information


**Supporting Information 1** Supporting File 1: Completed STROBE checklist for cohort studies.

**Supporting Information 2** Supporting File 2: ASM search terms, supplementary tables, and figures referenced in the text. S1: EHR antiseizure medicine search list; Table S1. ASM exposures and polytherapy; Table S2. Univariate linear regression analyses for individual ASMs with > 2 exposures.

## Data Availability

The data that support the findings of this study are available on request from the corresponding author. The data are not publicly available due to privacy or ethical restrictions.
