# Supplementary material for: Trends in Use of Antiseizure Medicines in Women With Epilepsy in Pregnancy During 2018–2023 in an Irish Maternity Hospital: A Cohort Study
Source: Obstet Gynecol Int. 2026 Sep 15;2026:9975717. doi: 10.1155/ogi/9975717 (PMC13575775; doi:10.1155/ogi/9975717)
Supplement: Supplementary file 1 [file OGI-2026-9975717-s001.docx]

**Supplemental File**

**S1: EHR anti-seizure medicine search list**

- brivaracetam
- cannabidiol
- carbamazepine
- cenobamate
- clonazepam
- eslicarbazepine
- ethosuximide
- fenfluramine
- fosphenytoin
- gabapentin
- lacosamide
- lamotrigine
- levetiracetam
- oxcarbazepine
- perampanel
- phenobarbital
- phenytoin
- pregabalin
- primidone
- retigabine
- rufinamide
- sodium valproate
- stiripentol
- tiagabine
- topiramate
- vigabatrin
- zonisamide

| **Table S1.** ASM exposures and polytherapy | |
| --- | --- |
|  | *ASM-exposed pregnancies*  *N = 286*  *n (%)* |
| ASM exposures  Levetiracetam  Lamotrigine  Clobazam  Carbamazepine  Zonisamide  Eslicarbazepine  Brivaracetam  Lacosamide  Pregabalin  Sodium valproate  Clonazepam  Ethosuximide  Oxcarbazepine  Topiramate  Gabapentin  Perampanel  Phenobarbital | 172 (60.1)  90 (31.5)  40 (14.0)  19 (6.6)  13 (4.5)  10 (3.5)  8 (2.8)  6 (2.1)  6 (2.1)  3 (1.0)  2 (0.7)  2 (0.7)  2 (0.7)  2 (0.7)  1 (0.3)  1 (0.3)  1 (0.3) |
| No. of ASMs in pregnancy  1  2  ≥ 3 | 215 (75.2)  57 (19.9)  14 (4.9) |
| *includes clonazepam, ethosuximide, oxcarbazepine, topiramate, gabapentin, perampanel, and phenobarbital | |

| **Table S2.** Univariate linear regression analyses for individual ASMs with >2 exposures | | | | |
| --- | --- | --- | --- | --- |
|  | *Intercept Estimate* | *Year Estimate* | *Year P-value* | *R-squared* |
| Brivaracetam | 1.333 | <0.001 | 1.000 | 0 |
| Carbamazepine | -1093.676 | 0.543 | 0.341 | 0.226 |
| Clobazam | -801.533 | 0.400 | 0.457 | 0.145 |
| Eslicarbazepine | -350.430 | 0.174 | 0.736 | 0.044 |
| Ethosuximide | 1.000 | 0.000 | N/A | N/A |
| Lamotrigine | -1485.943 | 0.743 | 0.439 | 0.156 |
| Lacosamide | 1013.000 | -0.500 | 0.667 | 0.250 |
| Levetiracetam | -1010.448 | 0.514 | 0.817 | 0.015 |
| Oxcarbazepine | 1.000 | <0.001 | N/A | N/A |
| Pregabalin | 1.000 | 0 | 0.158 | 0.532 |
| Sodium Valproate | 1.000 | 0 | N/A | N/A |
| Topiramate | 1.000 | 0 | N/A | N/A |
| Zonisamide | 801.442 | -0.395 | 0.029 | 0.840 |

**Figure S1.** Trends 2018-2023 for selected ASMs in WWE in pregnancy.
